# The Mosaic of “Seronegative” Antiphospholipid Syndrome

**DOI:** 10.1155/2014/389601

**Published:** 2014-03-17

**Authors:** Fabrizio Conti, Antonella Capozzi, Simona Truglia, Emanuela Lococo, Agostina Longo, Roberta Misasi, Cristiano Alessandri, Guido Valesini, Maurizio Sorice

**Affiliations:** ^1^Reumatologia, Dipartimento di Medicina Interna e Specialità Mediche, Sapienza Università di Roma, Viale del Policlinico 155, 00161 Roma, Italy; ^2^Dipartimento di Medicina Sperimentale, Sapienza Università di Roma, Viale Regina Elena 324, 00161 Roma, Italy

## Abstract

In the clinical practice it is possible to find patients with clinical signs suggestive of antiphospholipid syndrome (APS), who are persistently negative for the laboratory criteria of APS, that is, anti-cardiolipin antibodies (aCL), anti-**β**_2_-GPI antibodies and lupus anticoagulant. Therefore, it was proposed for these cases the term of seronegative APS (SN-APS). In order to detect autoantibodies with different methodological approaches, sera from 24 patients with SN-APS were analysed for anti-phospholipid antibodies using TLC immunostaining, for anti-vimentin/cardiolipin antibodies by enzyme-linked immunosorbent assay (ELISA), and for anti-annexin V and anti-prothrombin antibodies by ELISA and dot blot. Control groups of our study were 25 patients with APS, 18 with systemic lupus erythematosus (SLE), and 32 healthy controls. Results revealed that 13/24 (54.2%) SN-APS sera were positive for aCL (9 of whom were also positive for lysobisphosphatidic acid) by TLC immunostaining, 11/24 (45.8%) for anti-vimentin/cardiolipin antibodies, 3/24 (12.5%) for anti-prothrombin antibodies, and 1/24 (4.2%) for anti-annexin V antibodies. These findings suggest that in sera from patients with SN-APS, antibodies may be detected using “new” antigenic targets (mainly vimentin/cardiolipin) or methodological approaches different from traditional techniques (mainly TLC immunostaining). Thus, SN-APS represents a mosaic, in which antibodies against different antigenic targets may be detected.

## 1. Introduction

Antiphospholipid antibody syndrome (APS) is characterized by arterial and/or venous thromboses, recurrent abortions or foetal loss, and circulating antiphospholipid antibodies (aPL) [1, 2]. According to the classification criteria, diagnosis of APS requires the combination of at least one clinical and one laboratory criterion [3, 4]. Anti-CL and anti-*β*_2_-glycoprotein-I (a*β*_2_-GPI) antibodies, detected by enzyme linked immunosorbent assay (ELISA) and the lupus anticoagulant (LA) detected by clotting assays are the recommended tests for the detection of aPL [5, 6]. Nevertheless, in daily clinical practice it is possible to find patients with clinical signs suggestive of APS who are persistently negative for the routinely used aCL, a*β*_2_-GPI, and LA. Therefore, it was proposed for these cases the term of “seronegative APS” (SN-APS) [2, 7]. Three possible explanations for the existence of such “seronegative” cases have been proposed: either the diagnosis is wrong, or that previously positive aPL tests have become negative, or, as seems most likely, the current range of tests is inadequate [8].

Indeed, aPL represent a heterogeneous family of antibodies reacting with phospholipid-binding cofactor proteins, including not only *β*_2_-GPI [9, 10] but also different anionic phospholipids, proteins, or phospholipid-protein complexes, such as prothrombin [11], protein S [12, 13], protein C [14], annexin V [15], annexin II [16], oxidized low-density lipoprotein, lysobisphosphatidic acid (LBPA), and sulfatides [17–19].

Recently, with a proteomic approach, we identified vimentin/cardiolipin as a “new” target of the APS, also detectable in SN-APS patients [20]. In addition, we demonstrated the possibility of detecting aPL in SN-APS patients by immunostaining on thin-layer chromatography (TLC) plates [21, 22]. This latter nonquantitative technique identifies the reactivity of serum aPL with phospholipid molecules but with a different antigenic exposure as compared to ELISA method.

The aim of this study was to identify the best screening combination of “new” antigenic targets or methodological approaches to detect aPL in SN-APS patients.

## 2. Materials and Methods

### 2.1. Patients

The study included 24 consecutive patients, attending the Lupus clinic, Rheumatology Unit of the Sapienza University of Rome, presenting clinical features consistent with a diagnosis of APS but tested persistently negative (at least 2 times 12 weeks apart) for conventional aCL, a*β*_2_-GPI, and LA tests. Clinical manifestations included venous and/or arterial thrombosis and pregnancy morbidity as stated in the classification criteria for definite APS [3, 4]. Sera were collected at several times and stored at −20°C until use. Moreover, all patients showed normal screening for other causes of thrombophilia such as antithrombin, protein C and protein S deficiency, hyperhomocysteinemia, factor V, and prothrombin mutations.

Moreover, in this study, 43 consecutive out-patients, attending the Rheumatology Division of Sapienza University of Rome, were also included. Twenty-five had APS, diagnosed according to the Sapporo criteria [4]; they included both primary APS (*N* = 9) and APS associated with SLE (*N* = 16); 18 had SLE fulfilling the ACR revised criteria for the classification of SLE [23]. Finally 32 healthy subjects (normal blood donors) matched for age and sex were studied as controls.

This study was approved by the local ethic committees and participants gave written informed consent.

### 2.2. ELISA for aPL and Antiphospholipid-Binding Proteins

aCL and a*β*_2_-GPI ELISA kits were obtained from INOVA Diagnostics Inc. (San Diego, CA, USA). ELISA was performed according to the manufacturer's instructions. Antiannexin V and antiprothrombin were performed as previously described [11, 15] and confirmed by dot blot. A positive control and several normal human sera were run in the same assay to confirm the specificity of the results.

### 2.3. LA Test

LA was studied in two coagulation systems, a dilute sensitized activated partial thromboplastin time (aPTT) and a dilute Russell's viper venom time (dRVVT), followed by confirm test, using reagents and instrumentation by Hemoliance Instrumentation Laboratory, Lexington, MA, USA.

### 2.4. Detection of aPL by TLC Immunostaining

Immunostaining on TLC was performed as previously described, with slight modification [21, 22, 24, 25]. CL (Sigma Chemical Co., St Louis, MO, USA) and LBPA (Avanti Polar Lipids, Alabaster, AL, USA), 2 *μ*g, were run on aluminium-backed silica gel 60 (20 × 20) high performance thin layer chromatography (HPTLC) plates (Merck Co, Inc Darmstadt, Germany). Chromatography was performed in chloroform : methanol : CH_3_COOH : water (100 : 75 : 7 : 4) (v : v : v : v). The dried chromatograms were soaked for 90 sec in a 0.5% (w : v) solution of poly(isobutyl methacrylate) beads (Polysciences, Inc. Eppelheim, Germany) dissolved in hexane. After air-drying, the chromatograms were incubated at room temperature for 1 h with 1% BSA in PBS to eliminate nonspecific binding. The blocking solution was removed and replaced by a washing buffer (phosphate-buffered saline, PBS). The chromatograms were then incubated for 1 h at room temperature with sera, diluted 1 : 100 in the blocking solution. Sera were removed and chromatograms were washed 3 times for 10 min with PBS. Bound antibodies were visualized with HRP-conjugated goat anti-human IgG (Sigma-Aldrich, St Louis, MO, USA), diluted 1 : 1000 in 1% bovine serum albumin (BSA) in PBS, and incubated at room temperature for 1 h and immunoreactivity was assessed by the chemiluminescence reaction using the ECL Western blotting system (Amersham Pharmacia Biotech, Buckinghamshire, UK).

### 2.5. Detection of Antivimentin/Cardiolipin by ELISA

Anti-vimentin/cardiolipin complex antibodies were detected by a slight modification of ELISA method previously reported [20]. Polystyrene plates (96-well) were coated and incubated overnight at 4°C with 100 *μ*L/well of cardiolipin (50 *μ*g/mL; Sigma-Aldrich) in methanol and then with 100 *μ*L/well of human recombinant vimentin (5 *μ*g/mL; R&D System, Minneapolis, MN, USA) in 0.05 M NaHCO_3_ buffer, pH 9.5. Coated plates were incubated overnight at 4°C and then washed 3 times with phosphate-buffered saline containing 0.1% Tween 20 (PBS-T). Plates were blocked for 2 h at room temperature with 100 *μ*L of 1% BSA in PBS. After washing 3 times with PBS-T, the wells were incubated for 1 h at room temperature, with 100 *μ*L of patient sera, diluted 1 : 100 in the blocking buffer. Each serum was analyzed in triplicate. Goat polyclonal antivimentin (R&D Systems) was used as a positive control.

After 3 washes with PBS-T, the plates were incubated for 1 h at room temperature with horseradish peroxidase-conjugated antibodies; either goat anti-human IgG or rabbit anti-goat IgG (Sigma-Aldrich) was diluted in 1% BSA in PBS. The plates were washed 3 times with PBS-T; the bound peroxidase was then revealed with 100 *μ*L of* O*-phenylenediamine dihydrochloride and color development was stopped with H_2_SO_4_ 0.2 M for 5 min. Absorbance was measured at 492 nm in a microplate reader. Data were presented as the mean optical density (OD) corrected for background (wells without coated antigen). Thirty-two normal human sera were also tested, and a cut-off value was established at a mean of optical density (OD) ± 3 SD of normal human sera. Parallel experiments were performed in which all the procedures were identical without coated vimentin/cardiolipin complex. Virtually no reactivity was detected in all the samples (data not shown).

## 3. Results

### 3.1. Characteristics of Patients

All but one (a 39-year-old female with Asian ethnicity) SN-APS patients enrolled in this study were Caucasian, 18 females and 6 males with a mean age of 45.7 years (range 26–82), and a mean disease duration of 9.3 years (range 0.8–57). The clinical characteristics of SN-APS patients are reported in Table 1. APS patients (3 males and 22 females) showed a mean age of 43.9 years (range 27–71) and a mean disease duration of 9.6 years (range 0.1–34). SLE patients were all females with a mean age of 36.8 years (range 18–59) and a mean disease duration of 13.4 years (range 0.8–36). None of the healthy subjects experienced arterial or venous thrombosis nor recurrent fetal loss.

A statistically significant correlation was found between arterial and/or venous thrombosis and pregnancy morbidity in SN-APS (*P* < 0.001).

### 3.2. Detection of aPL by TLC Immunostaining

In SN-APS patients the results obtained by TLC immunostaining showed the presence of aCl in 13 out of 24 patients (54.2%); anti-LBPA antibodies in 9 out of 24 (37.5%). All sera positive for anti-LBPA were also positive for aCL.

In APS patients TLC immunostaining showed the presence of antibodies against CL in 17/25 (68%), against LBPA in 14/25 (56%). In SLE patients TLC immunostaining showed the presence of antibodies against CL in 11/18 (61.1%), against LBPA in 11/18 (61.1%). Finally, none of the healthy subjects showed aPL reactivity by TLC immunostaining (Table 2).

### 3.3. Detection of aPL and Antiphospholipid-Binding Proteins by ELISA

Eleven out of 24 SN-APS patients (45.8%) showed serum antibodies (IgG class) against vimentin/cardiolipin, 3 (12.5%) against prothrombin, and 1 (4.2%) against annexin V (Table 2). None resulted positive for antibodies against CL or *β*_2_-GPI.

In APS patients anti-vimentin/CL antibodies were detected in 22/25 (88%), antiprothrombin in 9/25 (36%), and antiannexin V in 14/25 (56%). Anti-CL reactivity was observed in 25/25 (100%). Anti-*β*_2_-GPI reactivity was observed in 18/25 (72%) APS.

In SLE patients anti-vimentin/CL antibodies were detected in 7/18 (38.8%), anti-prothrombin in 1/18 (5.5%), and antiannexin V in 4/18 (22.2%). Moreover, anti-CL reactivity was observed in 14/18 (77.7%) and anti-*β*_2_-GPI reactivity was observed in 7/18 (38.8%) SLE patients.

Finally, none of the 32 healthy subjects displayed positivity for the autoantibodies under test.

### 3.4. Associations between Autoantibodies and Clinical Features

Taken together, our findings show that in 19 out of 24 SN-APS (79.2%) at least one aPL/cofactor antibody was detected using the assays under test (Table 3).

Table 4 shows the prevalence of the autoantibodies in SN-APS patients with different clinical manifestations. No significant association was found between the prevalence of the clinical features in SN-APS patients and specific autoantibodies. The combination of two of the tested methodological approaches, TLC immunostaining for aCL and ELISA for anti-vimentin/cardiolipin complex antibodies, was able to detect aPL/cofactors in about two-thirds of SN-APS patients with thrombosis or pregnancy morbidity, with a small additional gain when also performing ELISA for prothrombin and annexin V (Table 4).

## 4. Discussion

In the clinical practice it is possible to find patients with clinical signs suggestive of APS, who are persistently negative for the routinely used assays to detect aCL, a*β*_2_-GPI and LA. For these cases the term of SN-APS has been proposed [26–30]. Similarly to classical APS, SN-APS can have an accelerated progression, resulting in multiorgan failure, ending to catastrophic APS [31].

Thus, since clinical features of SN-APS appear to be similar to APS, the most convincing explanation for the existence of such “seronegative” patients may be that the current range of tests is inadequate. It may depend either on limits of the traditional technical approaches or on the existence of different antigenic targets.

Considering the first possibility, we employed a different methodological approach for detection of aPL, TLC immunostaining, which relies upon the different partition characteristics of phospholipids between the surface (stationary phase) and mobile solvent phase for different solvent polarities. TLC immunostaining is useful for detection of aPL in the presence of cofactor proteins, mainly *β*_2_-GPI (provided with the medium). However, in this case, the binding of phospholipid to solid phase mainly involves both electrostatic and hydrophobic interactions. Thus, the antigen exposure is quite different as compared to that on the surface of microtitre wells, where phospholipids are coated in a layer of immobilized lamellar phospholipids [32]. Indeed, the present results obtained by TLC immunostaining showing the presence of aCL in more than half SN-APS patients (54.2%) confirm our previous reports [22] and suggest that this method may represent a useful tool to detect aCL, mainly in SN-APS patients. Moreover, all sera positive for anti-LBPA by TLC were also positive for CL. A possible limit of this method could be a relatively low sensitivity, since only 68% of “true” APS sera were positive for aCL by TLC.

Moreover, we have to consider that aPL represent a very heterogeneous family of antibodies because more than 30 different antibodies have been described in APS patients (the so-called autoantibody explosion in antiphospholipid syndrome) [33]. Among them, several “nonclassical” aPL are directed against platelets, glycoproteins, coagulation factors, lamins, mitochondrial antigens, and other cell surface markers [19]. With the aim to discover “new” antigenic targets of aPL, we identified, with a proteomic approach, vimentin/CL as a potential autoantigen in SN-APS patients [20]. In the present study we found serum antibodies against vimentin/CL in about 45% of SN-APS patients. Interestingly, these latter antibodies were detected in 4 SN-APS patients who were also negative for aCL by TLC immunostaining, supporting the view that antivimentin/CL test may be a relevant additional test for identification of aPL positive patients. Finally, we analyzed the SN-APS sera for the presence of antibodies against two major cofactor proteins for aPL, prothrombin and annexin V. Our results revealed that 3 out of 24 sera from SN-APS patients (12.5%) displayed antibodies to prothrombin and 1 (4.2%) to annexin V. The datum of antiprothrombin might be underestimated, since we tested anti-prothrombin antibodies in the absence of phosphatidylserine. Indeed, anti-phosphatidylserine/prothrombin antibodies, which have been recently standardized and validated [34], seemed to represent a stronger risk factor for thrombosis than antiprothrombin [35]. However, interestingly, in SN-APS group, anti-prothrombin antibodies were detected in 3 patients, which were negative for aCL by TLC immunostaining.

**Figure 1:**
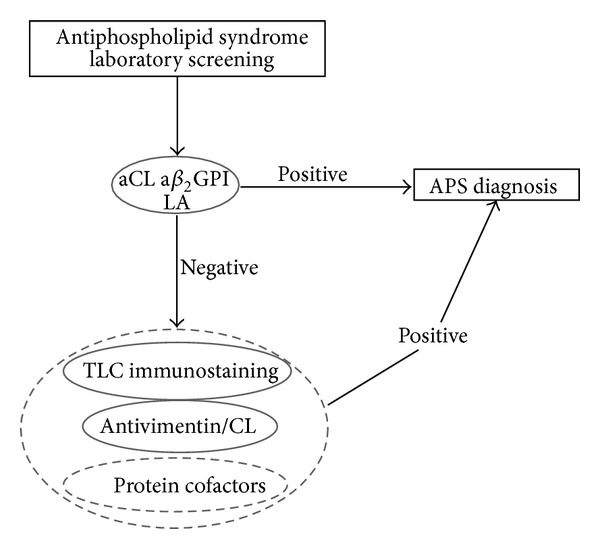
Proposed algorithm depicting the different diagnostic approaches in the most reliable sequence to reveal the hidden reactivity of the conventional aPL detection.

Taken together, these findings indicate that the execution of all these tests (TLC immunostaining, antivimentin/CL, antiprothrombin, and antiannexin V) can be very useful for identification of autoantibodies in so-called SN-APS patients. We can assert that, by using these approaches, it is possible to detect autoantibodies in the majority of the patients (79.2%). We suggest using all these different diagnostic approaches in the most reliable sequence, starting with TLC immunostaining, then antivimentin/CL ELISA, and eventually anticofactors ELISA to reveal the hidden reactivity of the conventional aPL detection (Figure 1). The combination of the first two diagnostic tests, TLC immunostaining and anti-vimentin/CL ELISA, detects aPL in about two-thirds of tested samples.

The presence of these antibodies has several implications. They might be used for early diagnosis of the syndrome, especially among patients at risk for thrombotic events and/or pregnancy morbidity. Moreover, autoantibody presence or absence might subclassify APS according to the association of these antibodies with clinical manifestations. At the end, they may help to identify patients who need secondary thromboprophylaxis with long-term anticoagulation, as well as prophylactic treatment during pregnancy.

However, although these approaches ameliorate our diagnostical possibilities, we are still unable to detect autoantibodies in a percentage of patients with a clinical picture suggestive of SN-APS. Other unidentified cofactors may be involved in sera reactivity. Further studies will shed light on “new” antigenic specificities in SN-APS as well as on the real importance of nonclassical antibodies found in SN-APS.

## 5. Conclusions

These findings suggest that in sera from patients with SN-APS, antibodies may be detected using “new” antigenic targets (mainly vimentin/cardiolipin) or methodological approaches different from traditional techniques (mainly TLC immunostaining). Thus, SN-APS represents a mosaic, in which antibodies against different antigenic targets may be detected.

## Figures and Tables

**Table 1:** Clinical characteristics of patients studied.

Characteristics *n* (%)	SN-APS (*n* = 24)	APS (*n* = 25)
Other autoimmune diseases	17 (70.8)	16 (64)
SLE	9 (37.5)	16 (64)
Sjögren syndrome secondary to SLE	2 (8.3)	8 (32)
Primary Sjögren syndrome	1 (4.2)	0
Rhupus	1 (4.2)	0
Mixed connective tissue disease	1 (4.2)	0
Undifferentiated connective tissue	1 (4.2)	0
Disease	1 (4.2)	0
Urticarial vasculitis	1 (4.2)	0
LED	1 (4.2)	0
Myasthenia gravis	0	1 (4)
None	5 (20.8)	9 (36)
Vascular thrombosis	18 (75)	24 (96)
Venous thrombosis	10 (41.6)	17 (68)
Arterial thrombosis	12 (50)	10 (40)
Recurrent thrombosis	8 (33.3)	10 (40)
Pregnancy morbidity	8 (33.3)	9 (36)
Normal fetus deaths	7 (29.2)	2 (8)
Premature births	0	0
Spontaneous abortions	5 (20.8)	8 (32)
Vascular thrombosis and pregnancy morbidity	3 (12.5)	6 (24)
Noncriteria APS features		
Livedo reticularis	5 (20.8)	7 (28)
Thrombocytopenia	1 (4.2)	5 (20)
Cognitive dysfunctions	1 (4.2)	6 (24)
Migraine	5 (20.8)	8 (32)
Seizures	0	5 (20)
Brain MRI scan abnormalities	10 (41.6)	8 (32)
Thrombotic risk factors	15 (62.5)	14 (56)
Hypercholesterolemia	5 (20.8)	10 (40)
Smoking	12 (50)	5 (20)
Hypertension	6 (25)	7 (28)
OC/HRT	2 (8.3)	2 (8)
Diabetes mellitus	1 (4.2)	0

**Table 2:** Occurrence of autoantibodies in SN-APS and control sera.

Autoantibodies	SN-APS (24) *n* (%)	APS (25) *n* (%)	SLE (18) *n* (%)	Healthy donors (32) *n* (%)
Anticardiolipin by TLC-immunostaining	13 (54.2)	17 (68)	11/18 (61.1)	0 (0)
Antivimentin/Cardiolipin	11 (45.8)	22 (88)	7/18 (38.8)	0 (0)
Antiprothrombin	3 (12.5)	9 (36)	1/18 (5.5)	0 (0)
Antiannexin V	1 (4.2)	14 (56)	4/18 (22.2)	0 (0)

**Table 3:** Positivity of autoantibodies in the 24 SN-APS sera.

Patient *n *	aCL by TLC immunostaining	Antivimentin/CL	Antiprothrombin	Antiannexin V
1	**Pos**	**Pos**	Neg	Neg
2	Pos*	**Pos**	Neg	Neg
3	Pos*	**Pos**	Neg	Neg
4	Pos*	Neg	Neg	Neg
5	**Pos**	**Pos**	Neg	Neg
6	Pos*	Neg	Neg	Neg
7	Pos*	Neg	Neg	Neg
8	**Pos**	**Pos**	Neg	Neg
9	Pos*	**Pos**	Neg	Neg
10	**Pos**	Neg	Neg	Neg
11	Pos*	**Pos**	Neg	Neg
12	Pos*	Neg	Neg	Neg
13	Pos*	Neg	Neg	Neg
14	Neg	Neg	Neg	Neg
15	Neg	Neg	**Pos**	**Pos**
16	Neg	Neg	**Pos**	Neg
17	Neg	**Pos**	**Pos**	Neg
18	Neg	Neg	Neg	Neg
19	Neg	**Pos**	Neg	Neg
20	Neg	**Pos**	Neg	Neg
21	Neg	**Pos**	Neg	Neg
22	Neg	Neg	Neg	Neg
23	Neg	Neg	Neg	Neg
24	Neg	Neg	Neg	Neg

*Also positive for anti-LBPA.

**Table 4:** Autoantibody prevalence in seronegative antiphospholipid syndrome (SN-APS) patients (*n* = 24) according to the clinical manifestations.

Autoantibodies (assay)	Total thrombosis (*n* = 18)	Arterial thrombosis (*n* = 12)	Venous thrombosis (*n* = 10)	Recurrent thrombosis (*n* = 8)	Pregnancy morbidity (*n* = 8)
aCL (TLC)	11 (61.1%)	7 (58.3%)	6 (60.0%)	5 (62.5%)	3 (37.5%)
Antivimentin/CL	7 (38.9%)	4 (33.3%)	5 (50.0%)	3 (37.5%)	5 (62.5%)
aCL (TLC) + antivimentin/CL	12 (66.7%)	8 (66.7%)	6 (60.0%)	5 (62.5%)	5 (62.5%)
Antiannexin V	0	0	0	0	1 (12.5%)
Antiprothrombin	1 (5.6%)	0	1 (10.0%)	1 (12.5%)	2 (25.0%)
aCL (TLC) + antivimentin/CL + anti-annexin V + antiprothrombin	13 (72.2%)	8 (66.7%)	7 (70.0%)	6 (75.0%)	7 (87.5%)
No autoantibodies	5 (27.8%)	4 (33.3%)	3 (30.0%)	2 (25.0%)	1 (12.5%)
